# A Recombinant Duck Plague Virus Containing the ICP27 Deletion Marker Provides Robust Protection in Ducks

**DOI:** 10.1128/spectrum.00983-23

**Published:** 2023-07-05

**Authors:** Ying Wu, Lu Liu, Mengya Zhang, Haichuan Zhan, Chenjia Wang, Mingshu Wang, Shun Chen, Renyong Jia, Qiao Yang, Dekang Zhu, Mafeng Liu, Xinxin Zhao, Shaqiu Zhang, Juan Huang, Xumin Ou, Sai Mao, Qun Gao, Di Sun, Bin Tian, Anchun Cheng

**Affiliations:** a Engineering Research Center of Southwest Animal Disease Prevention and Control Technology, Ministry of Education of the People’s Republic of China, Chengdu, People’s Republic of China; b International Joint Research Center for Animal Disease Prevention and Control of Sichuan Province, Chengdu, People’s Republic of China; c Key Laboratory of Animal Disease and Human Health of Sichuan Province, Sichuan Agricultural University, Wenjiang, People’s Republic of China; d Avian Disease Research Center, College of Veterinary Medicine of Sichuan Agricultural University, Wenjiang, People’s Republic of China; e Institute of Preventive Veterinary Medicine, Sichuan Agricultural University, Wenjiang, People’s Republic of China; Barnard College, Columbia University

**Keywords:** duck plague virus, UL54, ICP27, vaccine, virulence, *in vitro*, *in vivo*

## Abstract

Duck plague virus (DPV) is a member of *Alphaherpesvirus* genus and poses a major threat to waterfowl breeding. Genetic engineered vaccines that are capable of distinguishing naturally infected from vaccine-immunized animals are useful for eradicating duck plague. In this study, reverse genetics was used to develop an ICP27-deficient strain (CHv-ΔICP27), and its potential as a marker vaccination candidate was evaluated. The results showed that the CHv-ΔICP27 generated in this study exhibited good genetic stability *in vitro* and was highly attenuated both *in vivo* and *in vitro*. The level of neutralizing antibody generated by CHv-ΔICP27 was comparable to that induced by a commercial DPV vaccine, suggesting that it could protect ducks from virulent DPV attack. By using molecular identification techniques such as PCR, restriction fragment length polymorphism, immunofluorescence, Western blotting, and others, it is possible to differentiate the CHv-ΔICP27 from wild-type strains. Moreover, ICP27 can also be a potential target for the genetic engineering vaccine development of alphavirus or perhaps the entire herpesvirus family members due to the highly conservative of ICP27 protein in all herpesvirus family members.

**IMPORTANCE** The development of distinguishable marker vaccines from natural infection is a key step toward eradicating duck plague. Here, we generated a recombinant DPV that carries an ICP27 deletion marker that could be easily distinguished from wild-type strain by molecular biological methods. It was highly attenuated *in vitro* and *in vivo* and could provide comparable protection to ducks after a single dose of immunizations, as commercial vaccines did. Our findings support the use of the ICP27-deficient virus as a marker vaccine for DPV control and future eradication.

## INTRODUCTION

Duck viral enteritis (DVE), commonly referred to as duck plague (DP), is an acute infectious disease with high morbidity and mortality. It is caused by duck plague virus (DPV) and mainly infects ducks, geese, swans, and other Anseriformes birds of all ages. It is now one of the most serious threats to the duck industry (1). The clinical and pathological hallmarks of duck plague are edema of the head and neck, high fever retention, discharge of green loose stools, and obvious secretions around the eyelids. Ducks generally die after 1 to 5 days of displaying clinical symptoms (2, 3). According to the recent taxonomic classification by the International Committee on Taxonomy of Viruses (ICTV), DEV has been classified into the genus *Mardivirus*, subfamily *Alphaherpesvirinae* of the family *Herpesviridae* (4, 5). DPV has a linear double-stranded DNA genome and an icosahedral capsid that contains a nucleoprotein core surrounded by a membrane layer and a lipid envelope, similar to other alphaherpesviruses (6). DPV has a large genome with 78 genes (7), although most of these genes are currently unknown (8). ICP27 and its *Herpesviridae* homologs have RNA-binding and protein-interaction capabilities (9) that are involved in all stages of viral mRNA transcription, processing, export, and translation (10). ICP27 is also implicated in the regulation of nuclear protein quality control, cell cycle control, activation of stress signaling pathways, and apoptosis prevention (11–14). Currently, herpes simplex virus 1 (HSV-1) ICP27 is the best-studied member of this homolog in the *Herpesviridae* family (15, 16), followed by its homolog Kaposi’s sarcoma-associated herpesvirus (KSHV) ORF57 (17), human cytomegalovirus (HCMV) protein UL69 (18), pseudorabies virus (PRV) UL54 (19), Marek’s disease virus ICP27 (20), and bovine herpesvirus 1 ICP27 (21), etc.

Prior researches on ICP27 in HSV-1 discovered that ICP27 can both positively and negatively regulate the target genes expression (22, 23). Later research has demonstrated that ICP27 activates and represses of target gene expression at the posttranscriptional level of RNA (24), including splicing (25) and polyadenylation (26). Recent studies have identified the posttranscriptional activities of ICP27, including promoting viral RNA export (27) and stimulate translation of viral RNAs bound to it (28–30). In spite of the best-known function of ICP27 in mRNA processing, ICP27 of Marek’s disease virus has been found affect the horizontal transmission of the virus (20, 31, 32), whereas HSV-2 ICP27 can enhance the antiviral T cell response to improve the efficacy of gD- and gB-based vaccines (33, 34), implying its potential role in herpesvirus pathogenesis. Overall, since ICP27 in highly conserved in the herpesvirus family, all known herpesvirus homologs have been shown to share some functions of HSV-1 ICP27 (35).

The herpesvirus genes are known to be transcribed in the following order: immediate-early genes (α), early genes (β), and late genes (γ) (36, 37). Despite the fact that all herpesvirus subfamilies can encode ICP27, the its genotype varies among viruses. HSV-1 ICP27 has been identified as an immediate-early gene (15), and it interacts with various proteins, has a posttranscriptional modification function, and can regulate the expression of viral β and γ genes (16, 38), while the PRV UL54 gene has been identified as an early gene (19), and its role may be mainly involved in the viral DNA replication. HCMV UL69 in betaherpesviruses is an immediate-early gene that plays many roles during productive infection, including regulation of host cell cycle progression, regulation of viral gene expression, and nuclear export of intronless viral RNAs. However, studies have shown that substituting UL69 for ICP27 in HSV-1 cannot exert its function, indicating that its function is somewhat different from ICP27 in HSV-1 (39). EBV EB2 in gammaherpesviruses is also an immediate-early gene that may function by stabilizing mRNA and increasing translation (40). Despite the fact that KSHV ORF57 is a member of the gamma-herpes viruses, it is thought to be an early gene that promotes the accumulation and nuclear export of viral intronless RNA transcripts by interacting with mRNAs and cellular export proteins, as well as a viral splicing factor that regulates viral RNA splicing (41). In general, ICP27 is an immediate-early/early gene that either participates in viral DNA replication or regulates gene expression. Prior to viral DNA replication, it is transcribed (31, 42–47).

The ICP27 protein encoded by DPV UL54 gene was demonstrated to be one of three three immediate-early genes (48). Based on an amino acid sequence comparison, ICP27 was conserved across DPV and other herpesviruses. Functional domain prediction showed that DPV UL54 shares a conserved motif with the herpesvirus UL69 family, which includes HSV-1 ICP27, HCMV UL69, KSHV ORF57, and EBV EB2, etc. Previous studies found multiple nuclear localization signal (NLS) and nuclear export signal (NES) sequences in DPV ICP27, and the ICP27 protein is mainly located in the nucleus, which was attributed to its transcriptional regulation function (49). In addition, ICP27 in DPV is far less understood than its analog in human herpesvirus, particularly in terms of pathogenesis.

At present, timely vaccination of ducks with inactivated or attenuated DPV vaccines can effectively control the outbreak of duck plague. The development of a labeled vaccination that is both safe and effective will be a crucial step toward DPV eradication. Here, we constructed a DPV ICP27 gene deletion (CHv-ΔICP27) and a genetically revertant virus (CHv-ΔICP27R) to study their role in DPV pathogenesis, as well as their potential as a candidate labeled vaccine. The findings demonstrated that the CHv-ΔICP27 deletion marker could be stably inherited *in vitro*, and the loss of ICP27 significantly reduces the transcription of DPV virulence genes and thus impairs the virulence of CHv-ΔICP27 *in vitro* and *in vivo*. After a single dose of immunization, CHv-ΔICP27 provided ducks with protection comparable to that of a commercial vaccine (100% protection), and its safety compared to the wild-type strain was noticeably enhanced. CHv-ΔICP27 was differentiated from the wild-type virus using PCR, restriction fragment length polymorphism (RFLP) analysis, immunofluorescence analysis (IFA), and Western blotting (WB).

## RESULTS

### Construction and identification of CHv-ΔICP27 and CHv-ΔICP27R recombinant DPVs.

**FIG 1:**
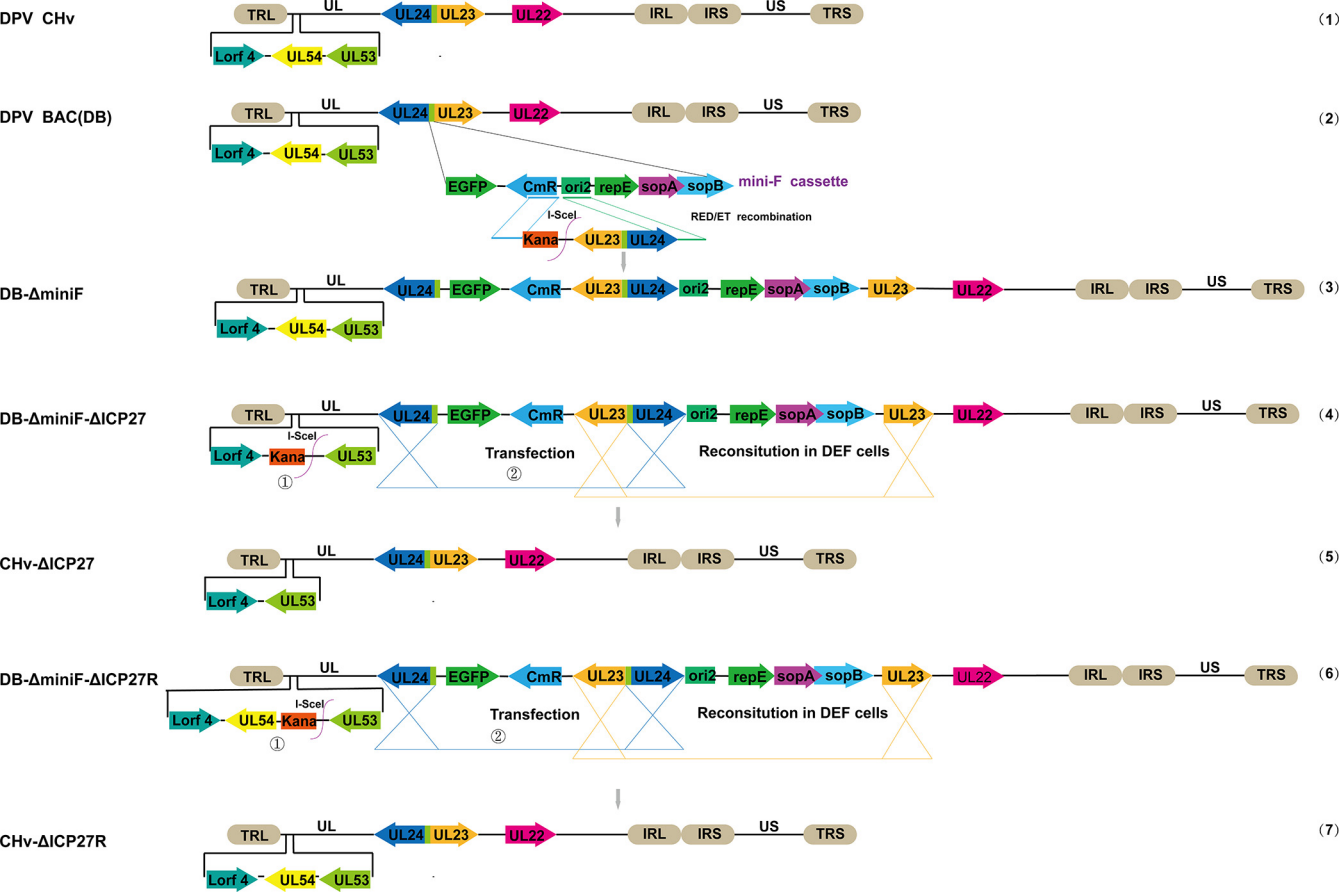
Schematic diagram of DPV-ΔICP27 and DPV-ΔICP27R construction process. Line 1, the structure of wild-type DPV (CHv strain) genome; line 2, genome structure of bacteria artificial chromosome of DPV (DPV BAC); line 3, BAC-based artificial chromosome genome DPV-BAC with miniF element removed; lines 4 and 5, the generation process of recombinant CHV-ΔICP27 virus. Step 1 occurs in GS1783 bacteria to obtain positive colonies of DB-ΔminiF-ΔICP27. After two rounds of RED recombination, the substituted Kana fragment of ICP27 will be scarless excised from DPV genome. Step 2 aims to rescue recombinant CHV-ΔICP27 virus. The reconstitution occurred in DEFs due to the duplicated sequence inside the miniF cassette. Lines 6 and 7, generation of CHV-ΔICP27R recombinant virus based on CHV-ΔICP27 by the same procedure.

**FIG 2:**
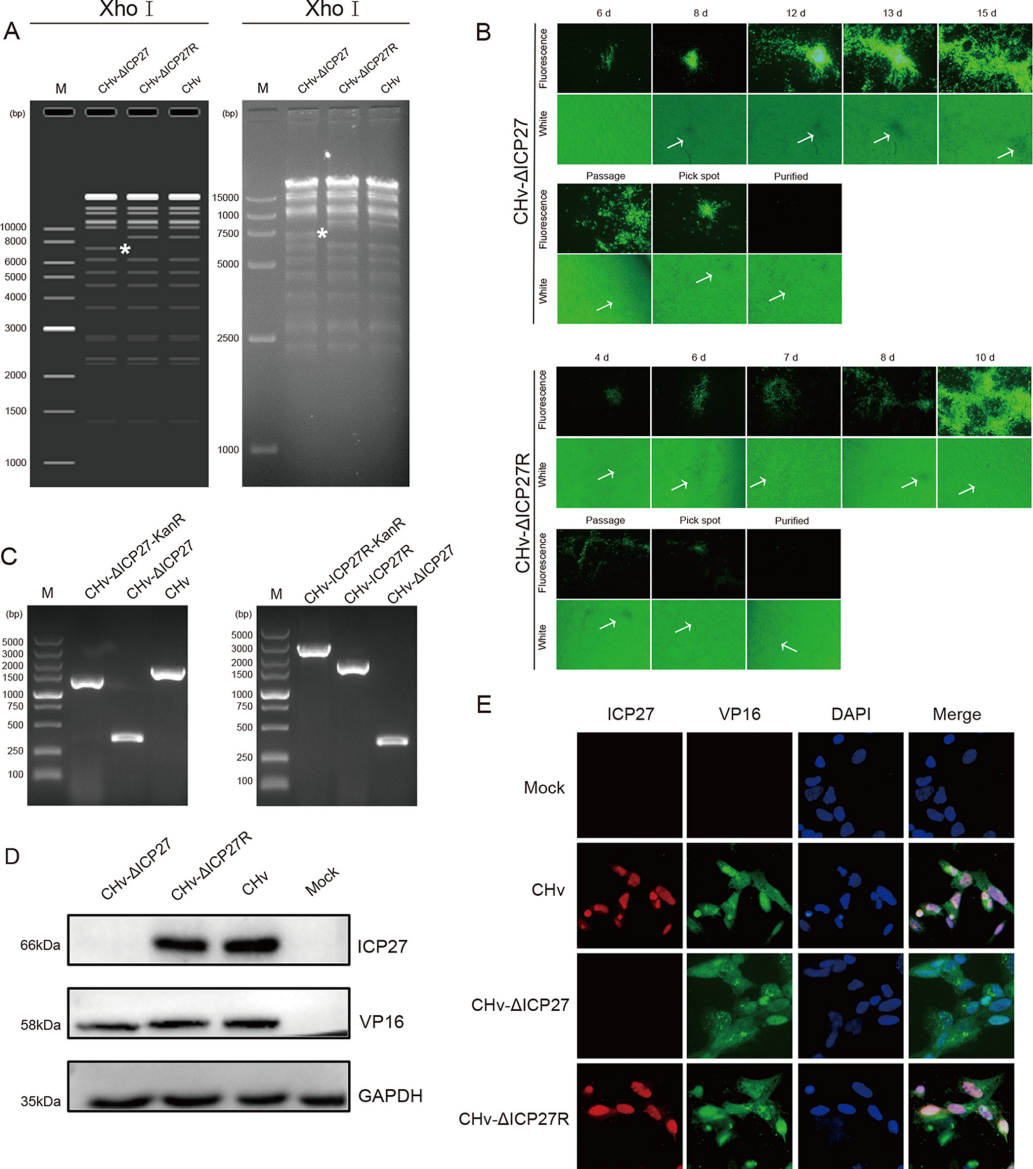
Rescue and identification of DPV-ΔICP27 and DPV-ΔICP27R. (A) RFLP results of the plasmid-level DB-ΔminiF-ΔICP27, DB-ΔminiF-ΔICP27R, and DB-ΔminiF following XhoI digestion. The asterisks indicated the specific band in DB-ΔminiF-ΔICP27 compared to that of wild-type and revertant virus. (B) Rescue and purification process of DPV-ΔICP27 and DPV-ΔICP27R in DEFs. The arrows indicate the cytopathic effect under white view. (C) PCR identification of DPV-ΔICP27 (left) and DPV-ΔICP27R (right). Lane 1 represents the successful replacement of the target gene fragment by the Kana fragment; lane 2 represents the PCR result of the positive bacteria that successfully removed the Kana fragment; lane 3 represents the unmodified parent strain. (D and E) Western blot and immunofluorescence analyses of DPV-ΔICP27 and ΔICP27R, with VP16 as a virus infection control.

We constructed CHv-ΔICP27 and CHv-ΔICP27R recombinant viruses based on the previously established DEV BAC (DB) backbone (50). In more detail, we first inserted the UL23-Kana fragment into the miniF element by homologous recombination, followed by arabinose-induced removal of the Kana selection marker via I-SceI restriction site. The plasmids of the chosen colonies in the aforementioned RED recombination were transfected into host cells to construct recombinant viruses free of any miniF cassette residue (Fig. 1, line 3). The ICP27 deficient strain CHv-ΔICP27 (Fig. 1, line 5) and its revertant strain CHv-ΔICP27R (Fig. 1, line 7) were subsequently generated. The obtained bacteria colonies were verified using RFLP analyses to ensure the plasmids of DB-ΔminiF-ΔICP27, DB-ΔminiF-ΔICP27R, and DB-ΔminiF were correctly generated. Compared to the parental and revertant strains, the ICP27-null virus showed a distinct band following XhoI digestion, which was exactly as predicted in the simulation diagram (Fig. 2A), demonstrating that the recombinant strains were successfully constructed. The plasmids DB-ΔminiF-ΔICP27 and DB-ΔminiF-ΔICP27R were then transfected into duck embryonic fibroblasts (DEFs) to rescue recombinant viruses. Green fluorescent spots that appeared in DEFs and gradually grew larger by 6 days posttransfection of DB-ΔminiF-ΔICP27 or 4 days posttransfection of DB-ΔminiF-ΔICP27R, as shown in Fig. 2B, indicate the successful rescue of recombinant viruses. Due to the inverted duplication of DPV genomic sequences within the miniF element, the miniF tag will progressively disappear during passage as a result of intracellular homologous recombination, resulting in stable and pure CHv-ΔICP27 recombinant virus that lacks all tags except the ICP27 deletion. The CHv-ΔICP27R was constructed as an experimental control in order to rule out nontarget mutations (Fig. 2B). PCR amplification of the regions flanking ICP27 confirmed that the successful deletion of ICP27 gene without any unexpected alterations (Fig. 2C). Furthermore, we analyzed the expression of ICP27 protein by Western blot and immunofluorescence assay. All three viruses, as expected, can express VP16 (Fig. 2D); however, CHv-ΔICP27 did not express the ICP27 protein. IFA also verified the lack of ICP27 protein in CHv-ΔICP27 (Fig. 2D and E). Moreover, infection with the ICP27-null virus appears to cause alterations in VP16 localization. We were unable to determine the varied subcellular location of VP16 in ICP27-deficient virus-infected cells either due to differences in the field of view we selected or to the nucleocytoplasmic shuttle function of ICP27 since we did not quantify the location variation of VP16. All of these studies collectively suggested that the ICP27-deficient virus and its revertant were successfully constructed and rescued.

### *In vitro* characterization and genetic stability of CHv-ΔICP27 and CHv-ΔICP27R.

**FIG 3:**
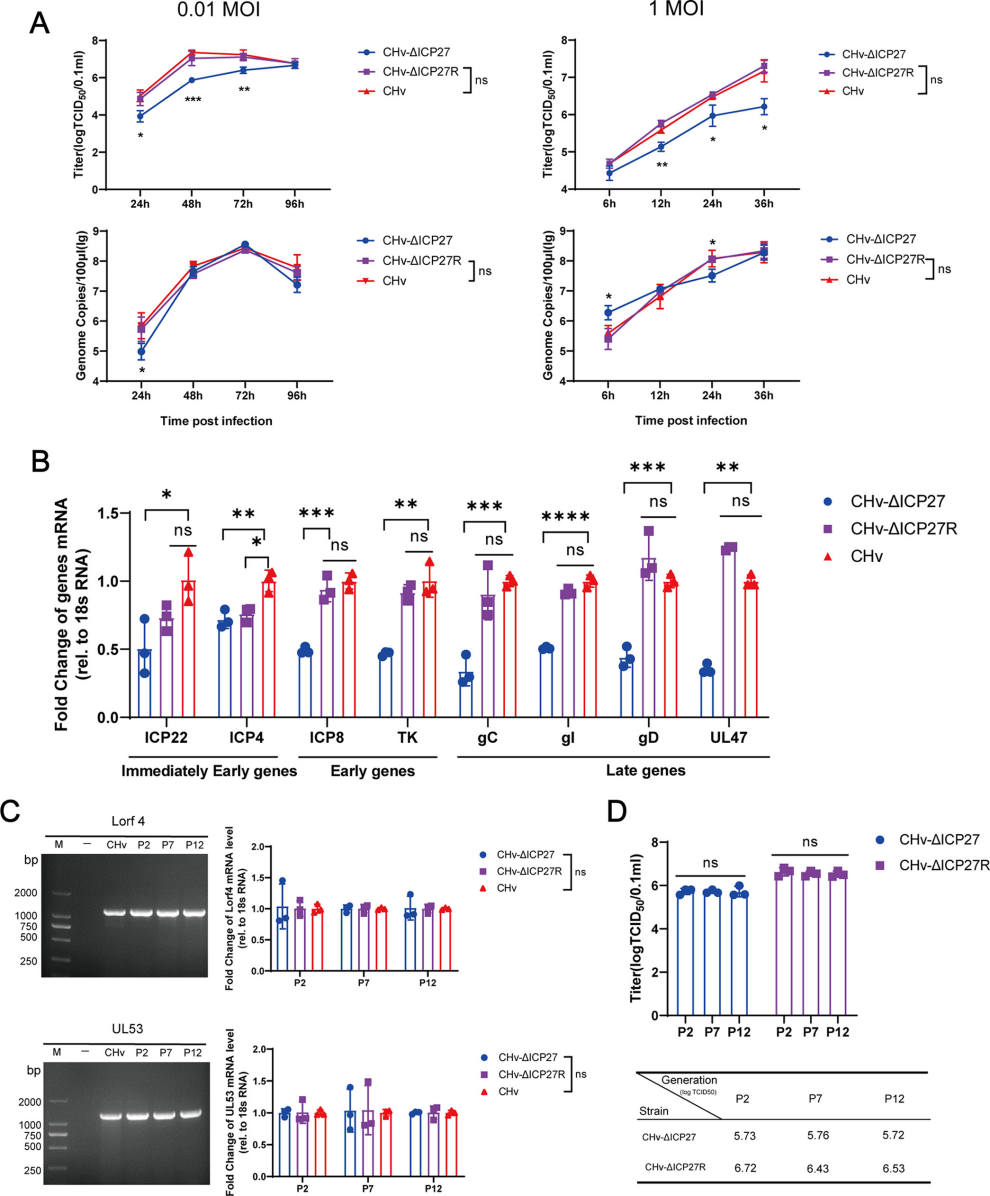
*In vitro* characterization and genetic stability of CHv-ΔICP27 and CHv-ΔICP27R. (A) Progeny virus production and genome replication in DEFs at an MOI of 1 or 0.01. (B) Effect of ICP27 deletion on DPV gene expression *in vitro* after respective virus infection at 1 MOI for 48 h. The relative abundances of DPV viral genes were measured by RT-qPCR normalized to duck 18S RNA. The results represent means, and error bars show the standard errors of the mean. Graphs represent data from at least three independent experiments. (C) Effect of ICP27 deletion on neighboring LORF4 and UL53 genes. The recombinant virus at P2, P7, and P12 were used as templates to test the expression of adjacent genes by PCR (left) and RT-qPCR (right). (D) The genome stability of corresponding recombinant viruses was ensured by determining the titer of the virus obtained at P2, P7, and P12.

To evaluate the effect of ICP27 loss on DPV replication *in vitro*, we infected DEFs with CHv-ΔICP27, CHv-ΔICP27R, and CHv at multiplicities of infection (MOIs) of 0.01 or 1 and then measured progeny viruses and genome replication at the indicated time points. Figure 3A demonstrates that in one-step or multiple-step growth curves, the absence of ICP27 significantly reduced the production of progeny DPV particles. However, the differences of CHv-ΔICP27 genome replication exist only at early stages of infection, and surprisingly, the genomes of CHv-ΔICP27 were even higher than that of wild-type CHv strains. It highlights that ICP27 may be important at various stages of the viral life cycle. To investigate the role of DPV ICP27 in viral gene transcription, the mRNA expression level of representative viral genes was tested 48 h after CHv-ΔICP27, CHv-ΔICP27R, or CHv infection at 1 MOI. As a result, the expression of all tested genes was significantly reduced, especially E and L genes, as well as the well-known virulence genes TK, gC, gI, and gD in herpesvirus, which could explain the impaired replication of recombinant virus *in vitro* (Fig. 3B).

To our knowledge, genes in herpesvirus genomes are tightly packed. We must be careful with the interpretation of gene deletion mutants, which might result in the abrogation of the expression of not only the deleted gene but also the neighboring genes due to the deletion of their promoters embedded in the body of deleted gene. We examined the genomic sequence and mRNA expression of the adjacent genes UL53 and LORF4 to rule out this possibility, which could hide the ICP27 phenotype. As expected, the genomic sequence and expression of LORF4 and UL53 remain comparable to wild-type CHv after 12 passages (Fig. 3C). The genetic stability of CHv-ΔICP27 and DPV CHv-ΔICP27R was tested at P2/P7/P12. As shown in Fig. 3D, the titers of CHv-ΔICP27 and CHv-ΔICP27R at different passages display no significant differences (*P* > 0.05). Overall, the results indicated that the deleted ICP27 would not be restored *in vitro* and that the genomes of CHv-ΔICP27 and CHv-ΔICP27R could be inherited stably after serial passing.

### Safety of CHv-ΔICP27 and CHv-ΔICP27R *in vivo*.

**FIG 4:**
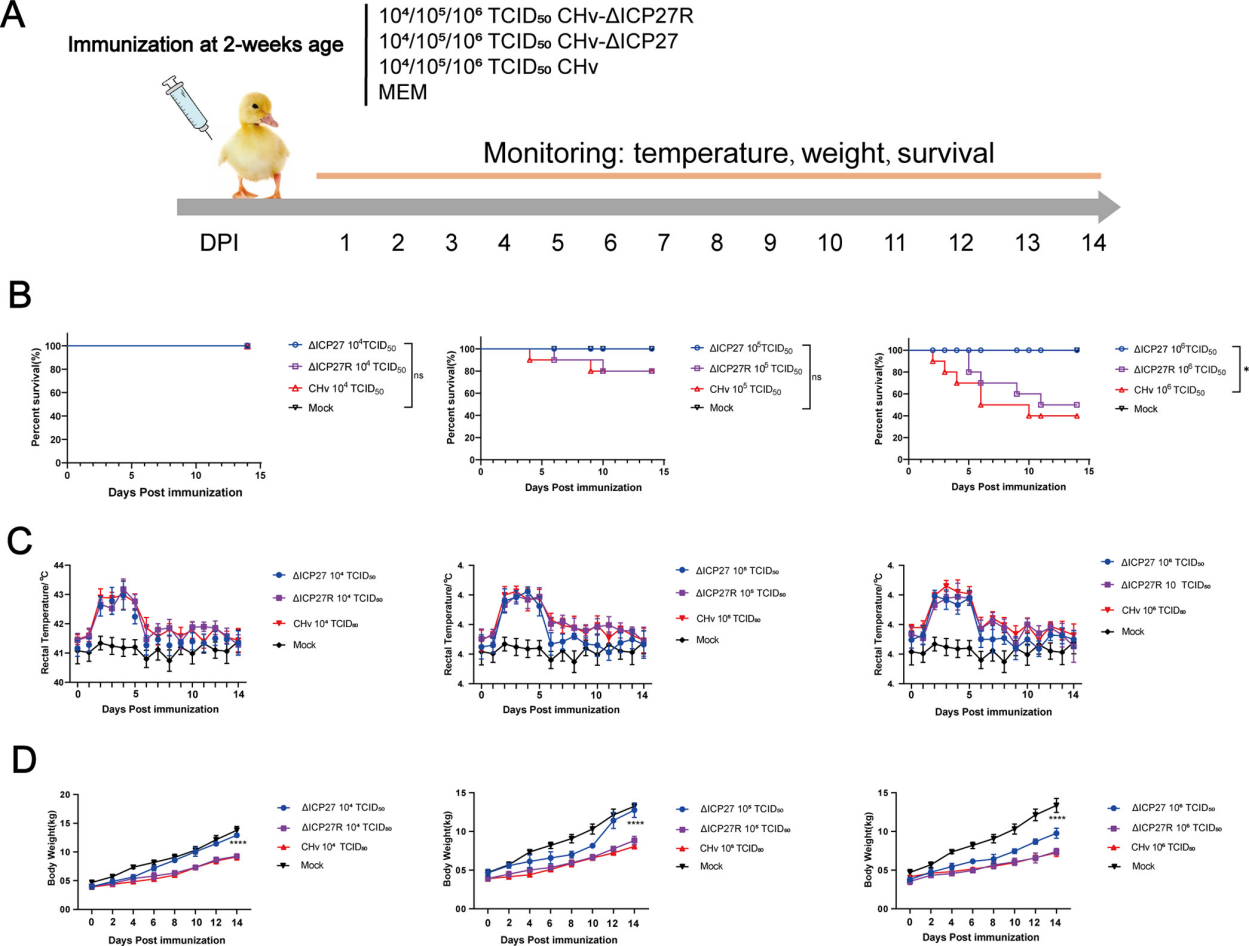
ICP27 contributes to the pathogenesis of DPV *in vivo.* (A) Flow chart of the animal experiment to study the virulence of DPV-ΔICP27 *in vivo*. Ten groups (10 ducks per group) of 14-day-old ducks were infected with MEM as a negative control or CHv, CHv-ΔICP27, or CHv-ΔICP27R strains (10^4^, 10^5^, or 10^6^ TCID_50_), respectively. (B to D) All inoculated ducks were evaluated to determine the survival rate (B), temperature (C), and body weight (D) for 2 weeks. Significance was determined by using a Mantel-Cox log-rank test (*, *P* < 0.05).

In order to investigate the pathogenicity of CHv-ΔICP27 and CHv-ΔICP27R *in vivo*, we injected every 10 ducklings with different doses (10^4^, 10^5^, or 10^6^ 50% tissue culture infective doses [TCID_50_]) of CHv-ΔICP27, CHv-ΔICP27R, or DPV CHv and monitored the temperature, weight, and survival of infected ducks until 14 days postinfection (dpi) (Fig. 4A). As a result, there were no deaths in the three gradients of the CHv-ΔICP27-immunized group, while the mortality of 10^6^ TCID_50_ in the CHv and CHv-ΔICP27R groups reached 60 and 50%, respectively (Fig. 4B). With the exception of the control group, subsequent temperature monitoring found that the body temperatures of ducks in all experimental groups rose to above 43°C in the peak period of 3 to 5 days at 10^4^, 10^5^, and 10^6^ TCID_50_ injections. However, at the end of monitoring period, the duck body temperatures returned to normal, i.e., between 40.5 and 42.5°C (Fig. 4C). Consistent with the temperature change trend following CHv and CHv-ΔICP27R inoculation, the body weights of ducks in the aforementioned two groups at different dose gradient were significantly lower than in the control group. In contrast, ducks inoculated with 10^4^ and 10^5^ TCID_50_ of CHv-ΔICP27 initially had decreased body weights but gradually recovered to body weights similar to those of the control group at 14 dpi, while 10^6^ TCID_50_ CHv-ΔICP27 injections caused ducks to lose significantly more body weight than did the control group (Fig. 4D).

**FIG 5:**
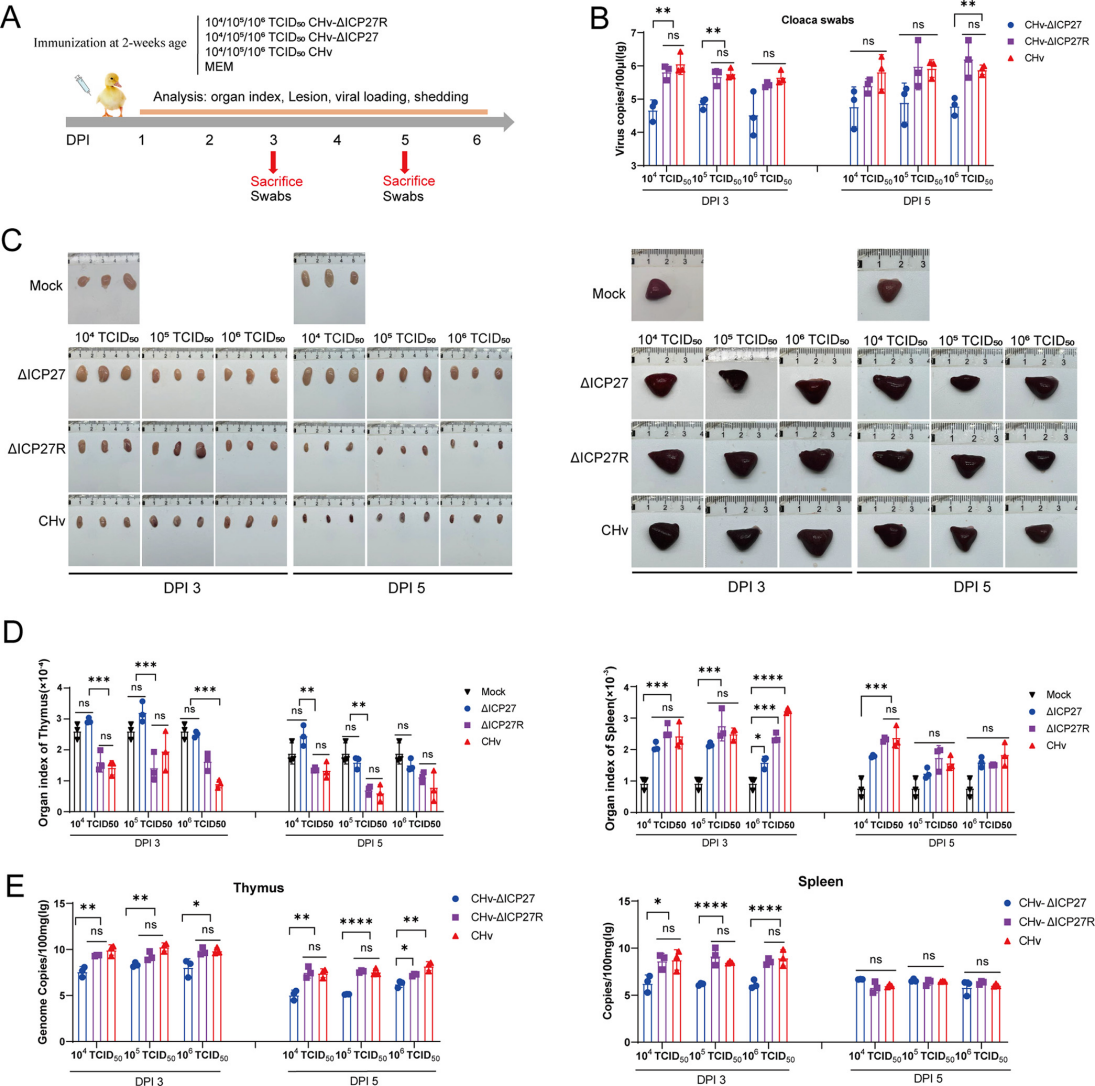
Tissue damage and viral load measurement in ducks. (A) Flow chart of the experiment to study the effect of DPV-ΔICP27 on tissue damage and viral loading. (Fourteen-day-old ducks were intramuscularly injected with CHv, CHv-ΔICP27, or CHv-ΔICP27R strains at a dose of 10^4^, 10^5^, or 10^6^ TCID_50_). Mock-infected ducks were used as negative controls. Three ducks in each group were sacrificed at 3 to 5 days postimmunization for lesions observation and sample collection. (B) Detection of virus shedding from cloaca at 3 to 5 dpi. (C) Observation of clinical lesions of the thymuses and spleens of ducklings in each group at 3 to 5 dpi. (D) Organ indexes of thymus and spleen of ducklings in each group at 3 to 5 dpi by organ weight/body weight ratio. (E) Detection of viral loads in the thymuses and spleens of ducklings infected with the indicated viruses.

To better characterize the virulence of CHv-ΔICP27 *in vivo*, we prepared another batch of ducks immunized with gradient dose of CHv-ΔICP27/CHv-ΔICP27R and CHV (Fig. 5A). Three ducks were slaughtered at random in each group to assess the lesions, organ index, viral loading, and shedding at 3/5 dpi. As shown in Fig. 5C, the thymuses of CHv- and CHv-ΔICP27R-infected ducks began to hemorrhage at 3 dpi, and the bleeding worsened at 5 dpi. Moreover, the thymuses of the CHv and CHv-ΔICP27R groups significantly atrophied compared to the control group and the CHv-ΔICP27 group. In contrast, 10^5^ and 10^6^ TCID_50_s in CHv-ΔICP27-infected ducks resulted in only minimal hemorrhage. Thymus organ index analysis confirmed the obvious thymus atrophy caused by CHv and CHv-ΔICP27R infection, while the thymus organ indices for CHv-ΔICP27-infected ducks showed no significant differences compared to the control (Fig. 5D). At 3 to 5 dpi, anatomical observation of spleens of the infected ducks in each group revealed that they were considerably swollen compared to the control group (Fig. 5C). The spleen organ index analysis findings concur with the anatomical observations (Fig. 5D). It seems the enlargement of the spleen with the ICP27-null virus at 10^4^ to 10^6^ TCID_50_ is similarly unavoidable. The viral loads determination of the target organs showed that ICP27 deficiency significantly reduced the viral loads in these two organs (Fig. 5E). In addition, ICP27 deletion dramatically decreased the viral shedding from cloaca (Fig. 5B). Taken together, these results revealed that the virulence of CHv-ΔICP27 was significantly attenuated in ducks, and 10^4^ TCID_50_ CHv-ΔICP27 is a safer immunization dose for ducks.

### Immune response to CHv-ΔICP27 immunization.

**FIG 6:**
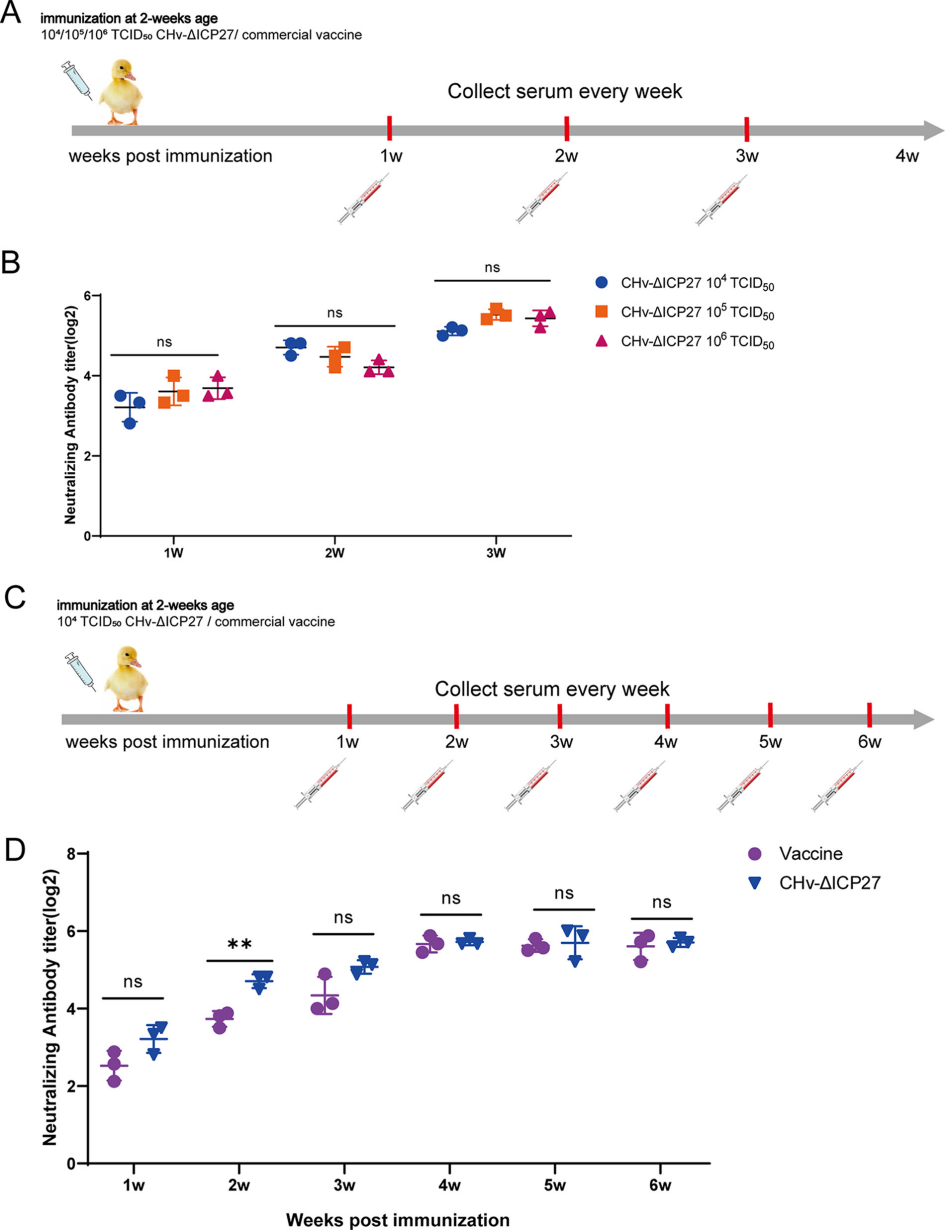
Immune response after DPV-ΔICP27 immunization. (A) Flow chart of the animal experiment used to test the induced antibody level by different titers of DPV-ΔICP27 immunization. Ten 14-day-old ducks were intramuscularly injected with 10^4^, 10^5^, or 10^6^ TCID_50_ DPV-ΔICP27, and sera from three ducks in each group were collected 1, 2, and 3 weeks postimmunization. (B) The neutralization antibody levels induced by different doses of DPV-ΔICP27 were tested and compared. (C) Flow chart of animal experiment compariing the induced antibody level by DPV-ΔICP27 and commercial vaccines. Fourteen-day-old ducks were intramuscularly injected with 10^4^ TCID_50_ CHv-ΔICP27 or commercial vaccines, and the sera from three ducks of each group were collected weekly for 6 weeks to test the immune response. (D) The neutralization antibody levels induced by 10^4^ TCID_50_ CHv-ΔICP27 and two-dose commercial vaccines were tested and compared.

Although ducks immunized with 10^4^ to 10^6^ TCID_50_ CHv-ΔICP27 showed 100% survival in the study described above, the degree of anatomical lesions and viral load varied. We looked at the amounts of neutralizing antibodies produced in groups infected with different doses of CHv-ΔICP27 to find a dose that would elicit an efficient immune response while still being safe (Fig. 6A). During our observation period, the results showed that there was no significant difference in neutralizing antibody levels among 10^4^ to 10^6^ TCID_50_ CHv-ΔICP27-immunized ducks. As shown in Fig. 6B, immunization with 10^4^ TCID_50_ CHv-ΔICP27 induced detectable neutralizing antibodies as early as 1 week postinfection; this increased at a similar rate and reached a level identical to the higher-dose immunization group within 3 weeks. This could explain the 100% survival rate of 10^4^ to 10^6^ TCID_50_ CHv-ΔICP27-immunized ducks. Further, we compared the level of neutralizing antibody induced by 10^4^ TCID_50_ CHv-ΔICP27 to that induced by DPV commercial vaccine in ducks (Fig. 6C). We found that the neutralizing antibody levels induced by 10^4^ TCID_50_ CHv-ΔICP27 immunization and vaccine were similar within 6 weeks, with the exception of 2 weeks postimmunization, when the CHv-ΔICP27 group was 4-fold higher than DPV vaccine immune group (Fig. 6D). Overall, we found 10^4^ TCID_50_ to be a safe and reliable dose for CHv-ΔICP27 immunization.

### Protective efficacy of a single-dose immunization with CHv-ΔICP27 in ducks.

**FIG 7:**
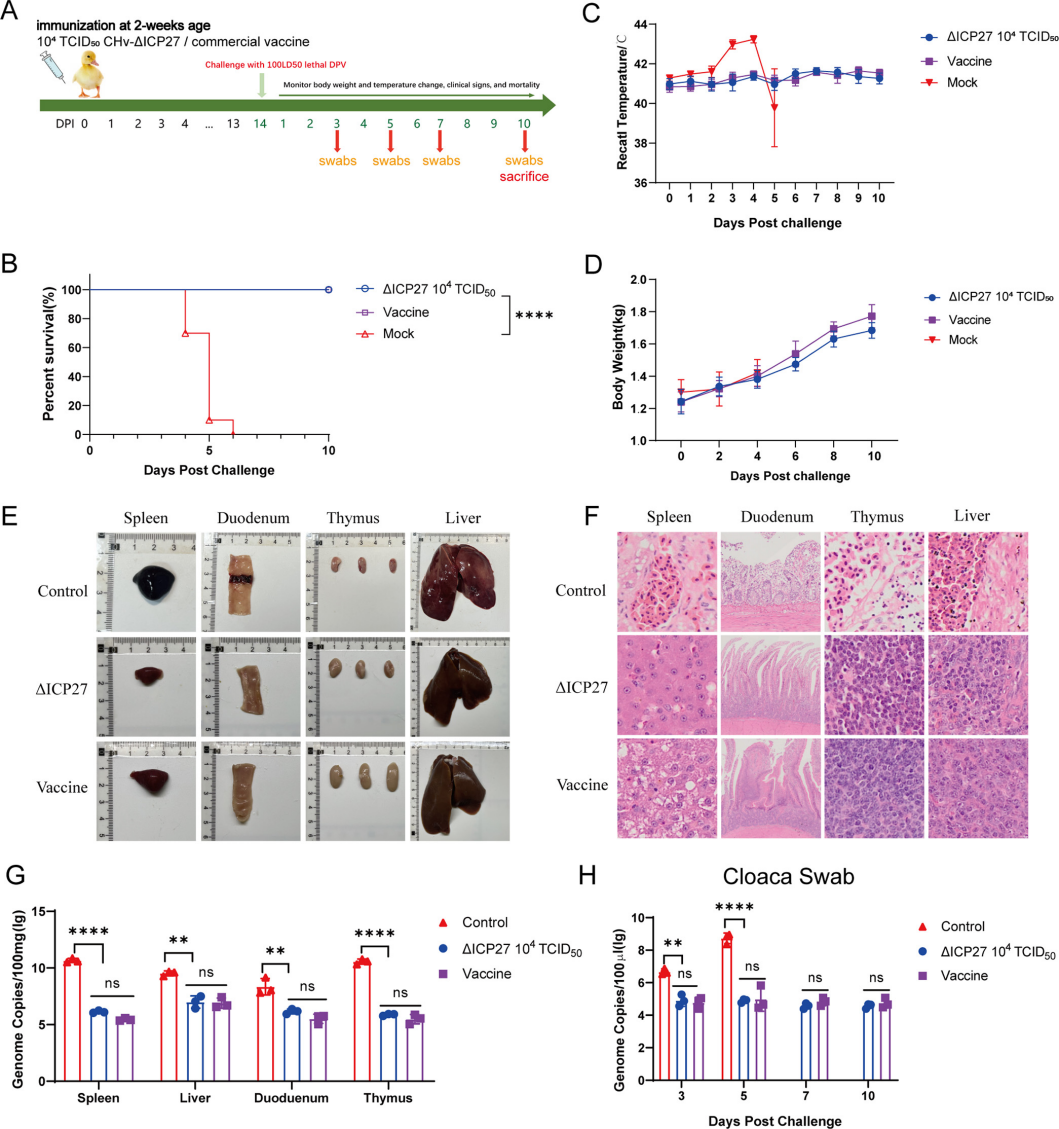
CHv-ΔICP27 protects ducks from virulent DPV challenge. (A) Experimental flow chart of the lethal DPV challenge test. Thirty ducks were randomly divided into three groups. Ten ducks in each group were immunized with 10^4^ TCID_50_ CHv-ΔICP27, commercial vaccine, or an equivalent volume of MEM before 100 LD_50_ lethal DPV challenge. (B) The deaths of ducklings in each group were recorded and plotted for 10 days postchallenge. The significance was determined by using a Mantel-Cox log-rank test (*, *P* < 0.05). (C and D) The body temperatures and weights were measured for 10 days postchallenge. (E) The clinicopathological changes in ducklings in each group were assessed by the size and lesions. (F) Microscopic lesions of ducklings after HE staining. (G) Viral load analysis of tissue samples after necropsy on day 10. The control group was sampled at the time of death. (H) Virus shedding test of cloacal swabs.

To explore whether the CHv-ΔICP27 could protect ducks from lethal challenge by virulent CHv. Thirty 14-day-old ducklings were divided into three groups: the 10^4^ TCID_50_ CHv-ΔICP27 immunization group, a commercially available vaccine control group, and a blank control group. As shown in Fig. 7A, the ducklings were challenged with 100 LD_50_ virulent DPV at 14 days to monitor the survival and morbidity after a single-dose CHv-ΔICP27 immunization. As a result, a single dose of 10^4^ TCID_50_ CHv-ΔICP27 may offer ducks the same level of defense against the lethal challenge of virulent DPV as the vaccine immunization. On the other hand, the blank control ducks all died between 4 and 6 days postchallenge (Fig. 7B). While the control ducks displayed rapid increasing body temperature, body weight and temperature monitoring revealed no significant differences between vaccine- and CHv-ΔICP27-immunized ducks (Fig. 7C and D). In contrast to vaccine- and CHv-ΔICP27-immunized ducks, which exhibited no overt clinical symptoms, ducks in the control group displayed fever, disheveled feathers, tears in their eyes, listlessness, reluctance to move when lying on the ground, heads retracted into their bodies, and insensitivity to external reactions.

Although CHv-ΔICP27 immunization allows ducks to maintain 100% survival from lethal challenge without exhibiting any clinical symptoms, it is unclear whether this immunization can completely clear the virulent virus from ducks and protect their organs from impairment. To investigate this further, the ducks inoculated with CHv-ΔICP27 and commercially DPV vaccine were sacrificed at 10 days postchallenge for lesion observation and viral load determination. Nonimmunized ducks that died after the challenge were autopsied and sampled for control. As shown in Fig. 7E, the organs of ducks immunized with CHv-ΔICP27 and vaccine had no obvious lesions. In contrast, the dead ducks in the control group had severe hemorrhage and swelling in the liver and spleen. Meanwhile, severe hemorrhage and typical bleeding ring symptoms occurred in the duodenum, intestinal villi decreased, the intestinal wall became thinner, and necrosis was visible in the intestine. Along with bleeding, control group thymuses shrank significantly more than for the CHv-ΔICP27 and vaccination groups. Further, hematoxylin and eosin (HE) staining of these tissues revealed no obvious histological or pathological changes in CHv-ΔICP27- or vaccine-immunized ducks. The spleen cells, on the other hand, were crushed and destroyed by pathogens in the control group without immunity, the nuclear membranes of some lymphocytes ruptured, and the nuclei were pyknotic. Red blood cells had accumulated in the pathological spleen. Thymus lymphocytes were severely pathologically damaged, decreased, fragmented, and disintegrated, and the number of normal cells was reduced. The major vein of the liver was also severely clogged with red blood cells. The borders between the hepatocytes were blurred, the hepatocyte nuclei were enlarged and rounded, a few nuclei were faintly stained or vanished, leaving nuclear shadows, and the cytoplasm exhibited increased eosinophilia and contained tiny granules (Fig. 7F). Viral load assessment in tissues and cloacal swabs of all ducks infected with CHv-ΔICP27 or vaccinated indicated no changes, suggesting that the CHv-ΔICP27 and vaccine had a clearance ability comparable to that of virulent DPV (Fig. 7G and H). In contrast, the viral load in the control group samples was significantly higher than that of the CHv-ΔICP27 and vaccine groups. Altogether, our findings imply that a single dose of CHv-ΔICP27 vaccination provided effective DPV protection comparable to the licensed DPV vaccine.

## DISCUSSION

Duck plague is one of the major infectious diseases that harm waterfowl farming (1). At the moment, the only commercial vaccinations approved for the protection of duck plague are inactivated vaccines and attenuated vaccines (51). There are no clinically authorized genetically engineered vaccinations, such as subunit vaccines, gene deletion vaccines, live vector vaccines, DNA-mediated vaccines, and synthetic peptide vaccines, although they are critical for the elimination of duck plague (52). Among these, gene deletion vaccines are one type of live attenuated vaccination that is thought to be closest to the pathogen’s mode of action and can more successfully generate humoral and cellular immunity (53). Here, we obtained a genetically stable vaccine candidate carrying an ICP27 deletion marker by using a reverse genetic manipulation method, and we found it can be quickly distinguished from wild-type virus by PCR, WB, IFA, and RFLP methods. Moreover, the ICP27-deficient DPV is highly safe and confers to ducks complete protection against lethal DPV attack, indicating its potential application as a labeled vaccine in controlling and eliminating DP outbreaks. In addition, due to the highly conservation of ICP27 among *Herpesviridae*, we believe this DPV has potential as a target for herpesvirus pathogenesis research and vaccine development.

The enormous genome of duck plague virus allows it to delete some nonessential genes and potential use in the development of vectored or labeled vaccines in the future. To test its possibility in developing labeled vaccine, we knocked out the conserved conserved immediate-early gene ICP27 to obtain an attenuated vaccine candidate. As a result, we found that the titer of CHV-ΔICP27 was much lower than that of its parental or revertant virus, whether in a one-step or a multistep growth curve, but there was less deficiency of genome replication during observation period. Similar results were obtained in other herpesviruses. The HSV-1 ICP27 mutant produced just 18% of the amount of viral DNA found in wild-type cells (54). In HSV-2, virus replication ability transfected with ICP27-targeted siRNA was significantly reduced at 12 to 24 h, but the subsequent changes were not significant (55). ICP27 mutants in PRV significantly reduce virus production but do not completely stop virus replication (56). We hypothesize that this may be because ICP27 has a greater effect on other steps of viral replication other than DNA synthesis. This makes sense because herpesvirus replication includes multiple steps, such as attachment, fusion, entry, uncoating, gene transcription, posttranscriptional modification, genome replication, protein synthesis, packaging, encapsulation, and release (57). Any of the blocked steps could impair the production of progeny viruses. Current findings on herpesvirus ICP27 homologs also support our interpretation. As a multifunctional protein, ICP27 homologs have been reported to be involved in viral transcription, translation, release, horizontal transmission, host mRNA processing, antiviral response, etc. (58–60). Our subsequent qPCR results further confirmed that ICP27 could promote virus replication by upregulating viral gene transcription. gC, TK, gD, gI, and UL47 are well-known virulence genes in alphaherpesviruses that participate in viral entry, assembly, cell-to-cell transmission, and maturation (61, 62), which could explain the attenuated virulence after ICP27 deletion. However, one seemingly contradictory result is noteworthy that Fig. 3A shows that viral genome replication of the ICP27-null virus is similar to that of wild-type virus, whereas Fig. 3B shows a substantial reduction in expression of many (early/late) viral genes, despite the fact that several early genes are involved in viral genome replication. As we know, the replication of the herpesvirus genome is carried out by the cooperation of the viral replication complex and host genes. Considering that we did not detect all replication-related genes here, it cannot be excluded that compensatory effects of other genes contribute to the absence of differences in genome replication between the ICP27-null and wild-type virus.

Another surprising finding in the *in vitro* experiment was that the level of ICP4 transcription was considerably reduced in the absence of ICP27. Given that ICP4 is a key transcriptional activator for the virus, the result is extremely harmful to viral transcription. As a result, we evaluated ICP4 protein expression and discovered that it was not affected by the absence of ICP27 (data not shown), demonstrating that ICP27 dynamically regulates ICP4 expression at the mRNA level. Previous research discovered that HSV-1 ICP27 is an activator of viral gene expression and can suppress ICP4 nuclear localization during virus infection (63). This could be one possible reason for ICP4 mRNA reduction after ICP27 deletion at the time we detected, since ICP4 is also a transcription repressor of itself and the transcription regulation function closely related to its nuclear location. Another factor may be the physical and functional interactions between ICP4 and ICP27, through which ICP27 could modulate the DNA-binding activity of ICP4 (64), and thus further influence its transcription regulation ability and expression. Another factor that should not be ignored is that the promoters of some herpesvirus genes are located within other genes, and gene knockout may lead to instability of the genome structure and impaired expression of adjacent genes, thereby masking the true phenotype of ICP27 gene deletion. Therefore, we examined the expression of adjacent genes and the *in vitro* stability of the deletion strain, and the results supported our previous findings that ICP27 knockdown directly caused a decrease in the titer of ICP27 deletion virus. At the same time, the high stability of the genome also indicates that the loss of ICP27 is irreversible.

A potential vaccine candidate is supposed to be safe and effective. In our study, the attenuation of DPV after ICP27 deletion was confirmed, since the clinical autopsy lesions, organ indexes, tissue viral load, and virus shedding of CHv-ΔICP27 was significantly reduced compared to that of CHv and CHv-ΔICP27R. It is important to note that none of the CHv-ΔICP27 dosage groups caused any duck deaths. However, the safety of CHv-ΔICP27 needs to be further improved, since each group of CHv-ΔICP27 ducks showed clinical symptoms such as fever and loss of body weight. Vaccine candidates should avoid fever as much as possible, which is also one of the important reasons that no genetically engineered vaccine currently for DPV has yet been approved. The lack of ICP27 alone is not mature enough as a vaccine at present, but fever caused by exogenous stimuli is a relatively common situation. In BoHV-1 (65) and PRV (66), there are cases of fever in vaccine research related to gene deletion. However, before it can be used as a vaccine candidate, the ICP27-deficient virus generated here needs to be significantly improved.

Despite the fact that there was no change in thymus index between CHv-ΔICP27- and mock-infected ducks, the spleen was enlarged as its parental and revertant virus, indicating that it still retains splenic, which is consistent with the general autopsy lesions. In terms of tissue viral load, the viral loads in the thymuses in CHv-ΔICP27-infected ducks were obviously lower than in CHv- and CHv-ΔICP27R-infected ducks. However, the viral loads in the spleen showed no difference at 5 dpi, which was consistent with the organ index trend. Another surprising finding was that the viral levels in the spleen were substantially lower than in the thymus. The atrophied thymus did not return to normal size during the 5-day observation period, whereas the splenic hyperemia was greatly reduced. This revealed that the organ index of thymus was much lower in the infected group than in the control group, while the spleen organ index was not significantly different from the mock-infected group. We thus hypothesized that the irreparable damage had severely weakened the thymus’ immune system, rendering it unable to fight off the infection. In the case of splenic congestion, the swelling had decreased greatly by day 5 after infection, and therefore the immune function had been restored, leading to the virus clearance from the spleen. It can be seen from the current data that after the deletion of ICP27, the low safety is still a problem worthy of further exploration. More experiments must be carried out in the future to reduce the incidence of fever and clinical symptoms, such as reducing the vaccine dose under the premise of ensuring an immune effect, or further deletion of virulence genes on this basis. Moreover, consider the horizontal transmission function of Marek virus ICP27 (20). Although it is a potential area for future research, we have yet to identify any genes in DPV that are specifically associated with horizontal transmission.

When detecting the regularity of neutralizing antibody production, we found that the immune response stimulated by CHv-ΔICP27 virus was stronger than the commercial vaccine in the first 3 weeks and then reached the same NT titer as that of the vaccine group. This may be attributed to the retained lower pathogenicity of ICP27-deficient virus, which thus confers on CHv-ΔICP27 stronger immunogenicity and leads to a stronger and faster immune response. It is also a benefit of attenuated labeled vaccines in various aspects. Moreover, the lethal DPV challenge experiment also revealed that CHv-ΔICP27 exhibited a protection efficiency comparable to commercial vaccines. Therefore, in addition to the role and mechanism of ICP27 in viral replication and pathogenicity, the feasibility of ICP27-deficient virus as a labeled vaccine is also worth investigating, but only if ICP27-deficient virus does not cause fever and clinical symptoms in immunized ducks. Moreover, this approach serves as a model for other herpesviral pathogenicity and genetic engineering vaccine research.

### Conclusion.

In general, we constructed an ICP27-deficient strain and discovered that it is genetically stable and distinguishable from wild-type DPV using multiple molecular identification methods, including PCR, RFLP, IFA, WB, etc. This strain is highly attenuated *in vitro* and *in vivo*, stimulating the production of high levels of neutralizing antibodies, and provides complete protection against virulent DPV attack with a single-dose immunization. This research may ultimately lead to the development of an effective DPV vaccine once its current associated virulence can be reduced.

## MATERIALS AND METHODS

### Cells and viruses.

DEFs were made from 7-day-old duck embryos and cultured in Dulbecco modified Eagle medium containing 10% newborn bovine serum according to previous studies. The DPV Chinese virulent strain (CHv; GenBank accession no. JQ647509.1) was preserved by our lab. DPV commercial vaccines were purchased from Harbin Pharmaceutical Group Biological Vaccine Company. The engineering host strain GS1783 was generously presented by Gregory A. Smith, Northwestern University. The pEPKans plasmid was generously provided by Klaus Osterrieder, Free University of Berlin, Berlin, Germany.

### Antibodies.

The primary antibodies used in this study, including the ICP27/ICP4 mouse polyclonal antibody and the VP16 rabbit polyclonal antibody, were produced by our lab and have been verified to be effective in previous studies. In brief, ICP4/27/VP16 recombinant proteins with C-terminal fusions of His tags were constructed using PET28a(+) as the vector and induced by IPTG (isopropyl-β-d-thiogalactopyranoside) in Escherichia coli before being purified by Ni-NTA sefinose resin 6FF (Sangon Biotech, catalog no. C600033). Then, 20 μg of purified ICP4 or ICP27 recombinant proteins was diluted 1:1 with the QuickAntibody-Mouse5W adjuvant (Biodragon, catalog no. KX0210041) and inoculated into mice on days 0 and 21, respectively. On day 35, the mouse polyclonal serum was collected. Rabbit polyclonal antibody against VP16 was prepared by a standard immunization procedure of three injections in 8 weeks according to the instructions of QuickAntibody-Rabbit8W adjuvant (Biodragon, catalog no. KX0210045). On days 0, 21, and 42, 50 μg of pure VP16 recombinant protein was administered, along with adjuvant, at a ratio of 1:1. On day 56, VP16 rabbit polyclonal antiserum was acquired. Mouse anti-GAPDH antibody (catalog no. 60004-1-Ig), horseradish peroxidase (HRP)-conjugated AffiniPure goat anti-mouse IgG(H+L) (catalog no. SA00001-1), and HRP-conjugated AffiniPure goat anti-rabbit IgG(H+L) (catalog no. SA00001-2) were purchased from Proteintech. The secondary antibodies used for IFA, including Alexa Fluor 488-conjugated goat anti-rabbit IgG(H+L) secondary antibody (catalog no. A11008) and Alexa Fluor 568-conjugated goat anti-mouse IgG(H+L) secondary antibody (catalog no. A11004), were purchased from Invitrogen.

### Generation and identification of CHv-ΔICP27 and CHv-ΔICP27R.

The CHv-ΔICP27 and CHv-ΔICP27R were constructed by utilizing the DPV-BAC bacterial artificial chromosome platform according to a previous study (50, 67). Briefly, the pEPKan-S plasmid was used as a template to amplify the Kana fragment. Then, the purified PCR product containing Kana and sequence flanking ICP27 gene was introduced to GS1783 bacteria containing the BAC-CHv ΔminiF genome to substitute the ICP27 gene. To get rid of Kana, a second round of RED recombination was carried out with 1% arabinose. After PCR identification, the positive colonies were designated as pBAC-CHv-ΔICP27 and extracted for further experiments. pBAC-CHv-27R was obtained by using the same procedure. The plasmids pBAC-CHv-ΔICP27 and pBAC-CHv-27R were subsequently transfected into DEFs to generate recombinant viruses. The miniF cassette could be self-excised after several rounds of passage due to the duplicated sequence inside the miniF cassette. PCR identifications were performed to confirm the deletion and restoration of ICP27 gene, as well as the genomic stability of its neighboring genes UL53 and Lorf4. Table 1 lists all of the identifying primers utilized.

**TABLE 1:** Primers and probes used in this study

Primer	DNA sequence (5′–3′)
ΔICP27-F	TTCCACGCCTACTTGTAATACGT
ΔICP27-R	TCCGTTCGTGAGCTATTAACATG
UL30-F	TTTTCCTCCTCCTCGCTGAGT
UL30-R	GGCCGGGTTTGCAGAAGT
UL30-probe	FAM-CCCTGGGTACAAGCG-MGB
ICP22-F	CGTAGCGTCACATCAAGCAG
ICP22-R	GCGTTTGGTCCCTATAACCTC
ICP4-F	AATCTATGCCCGTCCAAGCTC
ICP4-R	CCCGGACCCATTACTAGGCACA
ICP8-F	GGCAATACATGAAGATGGAG
ICP8-R	ATACAATGCAGAGTTGGTAC
TK-F	CCACCAGATATTACGCTCA
TK-R	CCAATAGAGTACTAAGGCTCA
gC-F	TCTTGGATCACAGGCCGAAC
gC-R	AGCTGCATACGCGACAGAAT
gI-F	CGAATCATAAAGGGCCGCATC
gI-R	ATTAGATCTCGTTACCCGCTTG
gD-F	GCCACGTCCAAAACAGCAAT
gD-R	GCCCAGTCAAGCCTATCCTC
UL47-F	AACGGAGTTGCTTGGAGAACA
UL47-R	AACGGAGTTGCTTGGAGAACA
Lorf4-F	GCCACCGGTTTAACTTTACC
Lorf4-R	GAGTACTTAGGATTCGCCAG
UL53-F	TCTGATTGTAGCCAATTCGC
UL53-R	TCATTTCGCCCACTCGCTAT

### RFLP.

The plasmids pBAC-DPV-ΔminiF/pBAC-DPV-ΔICP27/pBAC-DPV-ΔICP27R were extracted using a NucleoBond Xtra Midi kit (catalog no. 740410.50) according to the manufacturer’s instructions. Next, 1 μg of the plasmids listed above was digested with 4 μL of XhoI at 37°C for 20 min. The digestion products were loaded on a 0.8% agarose gel and run at 80 V for 20 min, followed by 120 V for 6 h. The results were recorded by an imaging instrument (Bio-Rad).

### Western blotting.

At 24 hpi, DEFs were infected with 1 MOI of CHv-DICP27, CHv-DICP27R, or wild-type CHv for Western blotting and ICP27 deletion studies. Mock-infected cells served as a negative control. The harvested cell precipitate was dissolved by addiing 100 μL of radioimmunoprecipitation assay buffer and 1 μL of phenylmethylsulfonyl fluoride. After being boiled for 10 min, the samples were loaded and separated using 10% SDS-PAGE. Next, proteins were half-dry transferred at 100 V for 1 h on polyvinylidene difluoride membranes (Bio-Rad, USA). The polyvinylidene difluoride membranes were incubated at 4°C overnight with 5% skimmed milk powder for blocking, followed by incubation with 1% bovine serum albumin-diluted mouse anti-ICP27 antibody (1:300), mouse anti-ICP4 antibody (1:500), mouse anti-VP16 antibody (1:800), or mouse anti-GAPDH antibody (1:5,000) at 4°C for one night. Before incubatiion of the membranes with HRP-labeled secondary antibody (1:5,000), they were washed with Tris-buffered saline/Tween (PBST) three times. Finally, the bands were visualized using an enhanced chemiluminescence chromogenic kit (Bio-Rad).

### Indirect immunofluorescence.

For IFA detection of recombinant viruses, infected DEFs grown on glass coverslips were plated in 24-well trays with CHv, CHv-ΔICP27, or CHv-ΔICP27R at an MOI of 0.01. Mock-infected cells were used as a negative control. In simple terms, we fixed the cells in each well with 1 mL of 4% paraformaldehyde at 4°C overnight and then washed the wells three times with PBST before adding 0.25% Triton X-100 for 30 min of permeabilization. Then, the cells were covered with blocking buffer (5% bovine serum albumin) at 4°C overnight before incubation with mouse anti-ICP27 antibody (1:100) and rabbit anti-VP16 antibody (1:200). The cells were then washed three times with PBST and incubated with TRITC/FITC-labeled goat anti-mouse/rabbit IgG antibody (1:1,000) at 37°C for 2 h. Finally, after a wash with PBST, 1 μL of DAPI was used to visualize nuclei, which were incubated at room temperature for 30 min. Images were observed and captured by using a fluorescence microscope (Nikon, Japan).

### RT-qPCR.

RT-qPCR was adopted to compare the mRNA expression of viral genes in CHv-ΔICP27, CHv-ΔICP27R and CHv *in vitro* and *in vivo*. RNA extraction of DEF and tissue samples was performed according to the instructions for the RNA-Easy isolation reagent (Vazyme, catalog no. R701-01). The obtained RNA was then reverse transcribed into cDNA using reverse transcription reagents (Yeasen, catalog no. 11141ES60). The cDNA from each group was used as a template for qualitative PCR amplification using the corresponding primers from Table 1. The relative expression level of each gene was calculated and normalized to 18S RNA. There were at least three replicates for the DEF or tissue samples.

### Characterization of CHv-ΔICP27 *in vitro*.

To characterize CHv-ΔICP27 *in vitro*, DEFs cultured in minimal essential medium (MEM) containing 10% newborn serum (NBS) were infected at an MOI of 0.01 (multistep growth curves) or 1 (one-step growth curves) with CHv-ΔICP27, CHv-ΔICP27R, or CHv. After 2 h of incubation, the medium was replaced with MEM containing 2% NBS for maintainance. The cultures were then collected at the indicated time points after infection and applied to subsequent titer and genome copy determinations. The collected samples were serially diluted (10^−1^ to 10^−8^) before being cocultured with fresh DEFs in 96-well plates to plot virus growth curves. Each dilution was repeated for eight wells. After 7 to 10 days of infection, the numbers of cytopathic wells of corresponding viruses were recorded to calculate the TCID_50_ values by using the Reed-Muench method (68). These samples were subjected to DNA extraction according to the manufacturer’s instructions to quantify the viral genome number. The genomic copy numbers of CHv-ΔICP27, CHv-ΔICP27R, or CHv were determined by the previously established TaqMan qRT-PCR method probing the UL30 gene and calculated using the following standard curve: *Y* = –4.262*X* + 43.675 (69).

### Animal experiment.

Fourteen-day-old ducklings were purchased from a farm run by Sichuan Agricultural University (Sichuan, China). The experimental ducklings were DPV negative or DPV antibody negative. The experimental animal protocol was approved by the Ethics and Animal Welfare Committee of Sichuan Agricultural University and carried out in accordance with the Chinese version of the *Guide for the Care and Use of Laboratory Animals*. To evaluate the safety of CHv-ΔICP27, 100 14-day-old ducklings were randomly divided into 10 groups and intramuscularly injected with 1 mL of CHv-ΔICP27 (10^4^, 10^5^, and 10^6^ TCID_50_/mL), CHv-ΔICP27R (10^4^, 10^5^, and 10^6^ TCID_50_/mL), CHv (10^4^, 10^5^, and 10^6^ TCID_50_/mL), or MEM, respectively. The rectal temperatures and survivals of ducks in each group were recorded every day, while the body weights of ducks were recorded every 2 days. To better evaluate the virulence of CHv-ΔICP27 *in vivo*, another batch of ducks was inoculated with the same dose of these viruses, and three ducklings in each group were killed at 3 to 5 days postinoculation to observe the pathological changes of the organs. Meanwhile, the organ and body weights of sacrificial ducks in each group were recorded and used to calculate the organ index. The viral loading and shedding in ducks after recombinant virus inoculation were determined by quantification of the DNA copies in each organ and cloacal swab. Sera from ducks injected with 10^4^, 10^5^, and 10^6^ TCID_50_/mL CHv-ΔICP27 were collected at 1, 2, and 3 weeks postimmunization for neutralization antibody titration to screen a safe but effective dose for subsequent immunization. Then, twenty 14-day-old ducks were randomly divided into two groups and immunized with 10^4^ TCID_50_ CHv-ΔICP27 or two doses of commercial vaccine to compare the neutralizing antibody production.

The same batch of ducks that had been evaluated for safety was immediately used to test for protective efficacy against challenge to evaluate the protective efficiency of CHv-ΔICP27. Ducklings were challenged with 100 LD_50_ lethal DPV through intramuscular injection 14 days after receiving 104 TCID_50_ of CHv-ΔICP27, commercial vaccination, or MEM immunization. After challenge, we observed and recorded the body temperatures, weights, and deaths of ducks in each group at the indicated time points. Cloacal swabs were collected on days 3, 5, 7, and 10 postchallenge to detect virus shedding, and necropsy samples were obtained when the ducks died. The remaining ducks were necropsied and sampled on day 10 after challenge. Meanwhile, the obtained tissues were sliced and stained with HE to assess the degree of tissue injury.

### Quantification of viral loads *in vivo*.

To quantify the copy numbers of the DPV genome *in vivo*, the DNA extracted from collected tissue and swab samples was used as templates to evaluate the clearance of lethal virus after immunization. Briefly, viral DNA was first extracted from infected animal tissues; then, 100-mg tissue samples were crushed into powder using a homogenizer and grinding balls before isolation with DNAiso reagent. The DPV genome copy numbers in these samples were then determined by using TaqMan qRT-PCR as previously described, with UL30 gene-specific probes and primers, and calculated from a previously generated standard curve: *Y* = −4.262*X* + 43.675 (69).

### Statistical analysis.

All data were subjected to a Student *t* test using GraphPad Prism 5.0. The results are presented as means ± the standard deviations (SD). Asterisks indicate *P* values (*, *P* < 0.05; **, *P* < 0.01; ***, *P* < 0.001), and “ns” means not significant (*P* > 0.05). *P* values of <0.05 were considered significant.
